# Changes in prevalence and predictors of tobacco smoking and interest in smoking cessation in Turkey: Evidence from the Global Adult Tobacco Survey, 2008–2016

**DOI:** 10.18332/tpc/152748

**Published:** 2022-09-19

**Authors:** April D. Summers, Hulya Sirin, Krishna Palipudi, Toker Erguder, Angela Ciobanu, Indu B. Ahluwalia

**Affiliations:** 1 Office on Smoking and Health, Centers for Disease Control and Prevention, Atlanta, United States; 2 University of Health Sciences, Gulhane School of Medicine, Ankara, Turkey; 3 World Health Organization Country Office, Ankara, Turkey; 4 World Health Organization Regional Office for Europe, Copenhagen, Denmark

**Keywords:** smoking, thinking about quitting, sociodemographic characteristics, tobacco

## Abstract

**INTRODUCTION:**

Turkey conducted three rounds of the Global Adult Tobacco Survey (GATS) in 2008, 2012, and 2016 to monitor tobacco use and key tobacco control indicators. The prevalence estimate of adult tobacco use was 31.2% in 2008 and it declined to 27.1% in 2012.

**METHODS:**

GATS is a nationally-representative, cross-sectional household survey of tobacco-use and related behaviors among adults aged ≥15 years. Outcome measures were prevalence of current tobacco smoking and interest in quitting smoking. Multivariable logistic regression analyses assessed changes in the adjusted prevalence and predictors of the outcome variables.

**RESULTS:**

The unadjusted prevalence of tobacco smoking among adults was 31.6% in 2016; a significant increase in the adjusted prevalence of 4.5% from 2012 to 2016. A significant 19.4% decline was observed in interest in quitting smoking from 2012 to 2016. Tobacco smoking was lower among women (adjusted prevalence ratio, APR=0.38) and rural residents (APR=0.79), and higher among adults aged 25–64 years compared to those aged 15–24 years (APR=1.63), and those who lived with other adults who smoke tobacco (APR=1.55). Predictors of increased interest in quitting smoking included rural residence (APR=1.13), higher education level (APR=1.21–1.36), awareness of anti-tobacco warnings and advertisements (APR=1.30), and belief that smoking causes severe health consequences (APR=1.57).

**CONCLUSIONS:**

This study identified opportunities to reduce tobacco smoking and increase interest in quitting, including increasing awareness of the health consequences of smoking and of evidence-based cessation resources. This study highlights Turkey’s commitment to assessing or monitoring tobacco use and key tobacco indicators to inform their policies and programs in a changing tobacco landscape.

## INTRODUCTION

In 1996, Turkey passed the Law on Prevention of Hazards of Tobacco Products Number 4207, which launched their tobacco control efforts by prohibiting smoking in some public places, such as indoor areas of public workplaces, and on public transport, prohibited some advertisement and promotion of tobacco products, and prohibited the sale of tobacco products to youth aged <18 years^1^. This was followed by Turkey’s ratification of the WHO Framework Convention on Tobacco Control (FCTC) in 2004^2^, which signified their agreement and commitment to implementing recommended MPOWER measures (six evidence-based tobacco control measures). In line with the WHO FCTC and MPOWER, in 2006 Turkey developed a National Tobacco Control Program and Action Plan with ten articles of action items for implementing tobacco control and cessation policies and programs, including monitoring and evaluation of tobacco use^1^.

As part of their national action plan, Turkey monitors tobacco use and key tobacco control measures on regular intervals through implementing several national surveys, including GATS. Turkey was the first country to conduct three rounds (2008, 2012 and 2016) of the Global Adult Tobacco Survey (GATS) and has conducted several other national surveys that include questions on tobacco use, such as the Health Interview Survey (HIS)^3^. From 2008 to 2012, a significant decrease in the prevalence of current tobacco smoking of 4.1% was observed in GATS data^4^. Additionally, data from GATS 2008 and GATS 2012 provided evidence that the proportion of current tobacco smokers contemplating smoking cessation significantly increased in Turkey from 21.2% in 2008 to 26.9% (p<0.01) in 2012^5^.

However, the significant decrease in the prevalence of tobacco smoking in Turkey observed between 2008 and 2012 did not appear to be sustained in the GATS 2016 survey. In the only study to date that included data from the three rounds of Turkey GATS surveys, Ahluwalia et al.^6^ reported a significant increase in the unadjusted prevalence of current tobacco smoking of approximately of 4.5% between GATS 2012 and GATS 2016, and an increase of 0.4% between GATS 2008 and GATS 2016.

Although few studies have examined the prevalence or impact of tobacco smoking in Turkey after 2012, there is some evidence that the burden of tobacco smoking remains high: in data from the HIS survey in 2019, the overall prevalence of tobacco smoking among adults aged ≥15 years was 31.3%^3^, and in 2017 an estimated 77000 deaths in Turkey were attributed to tobacco smoking and 11000 deaths were attributed to secondhand smoke exposure among non-smokers^7^. Documenting changes in the prevalence over time and investigating sociodemographic predictors of tobacco smoking and interest in quitting smoking are important for informing future surveillance and targeted tobacco control interventions. Because the data collected in GATS allow for more in-depth exploration of the patterns and predictors of tobacco smoking and interest in smoking cessation than are collected in the other national surveys in Turkey, and for comparability between survey years, we conducted an analysis limited to the three rounds of GATS Turkey data from 2008 to 2016. The objectives of the present analysis included examining changes in the prevalence of current tobacco smoking and interest in quitting smoking between survey years, and assessing predictors of current tobacco smoking and interest in quitting smoking.

## Methods

### Data source

GATS is a nationally-representative, cross-sectional survey of non-institutionalized adults aged ≥15 years, conducted using standardized protocols and data collection instruments^8^. Selected survey respondents live in households and are interviewed in-person by a trained interviewer with a questionnaire of tobacco-use and other tobacco control indicators. The Turkey GATS in 2008, 2012, and 2016 used a multi-stage, geographically clustered sample design; sample weights were calculated for each survey that allowed for the computation of weighted estimates that were representative of the overall population, as well as by gender^8^. The 2008 and 2012 sample clusters were also stratified by urban/rural residence; in the 2016 survey, selected clusters were determined without distinction between urban and rural residence. One adult from each participating household was randomly selected to complete the survey.

Overall, response rates were 90.9% in 2008, 90.1% in 2012, and 82.2% in 2016. Household response rates were 93.7%, 86.5% and 83.2% in 2008, 2012, and 2016, respectively; individual response rates were >97% each survey year. In total, the final sample size for each round of GATS Turkey was 9030 persons (weighted n=51.2 million) in 2008, 9851 persons (weighted n=54.5 million) in 2012, and 8760 persons (weighted n=60.9 million) in 2016.

### Measures

The dependent variables were current tobacco smoking and interest in quitting tobacco smoking. Current tobacco smoking was defined as daily or less than daily smoking, based on the response to the question: ‘Do you currently smoke tobacco on a daily basis, less than daily, or not at all?’. Interest in quitting tobacco smoking was assessed among current tobacco smokers, and was defined as planning to quit within the next month, thinking about quitting within the next 12 months, or intending to quit someday, but not within the next 12 months; a response of either ‘not interested in quitting’ or ‘don't know’ were considered not to be interested in quitting smoking.

Independent variables included year of the survey, sex, age (15–24, 25–44, 45–64, and ≥65 years), residence (urban/rural), education level (no formal/less than primary; primary/less than secondary; secondary or high school; college/university degree or higher), wealth index (quintiles), awareness of anti-tobacco warnings and advertisements on media (health warning labels on cigarette packages; billboards; newspapers or magazines; radio; and local television), awareness of any advertisements promoting tobacco products, belief in smoking as a cause of severe health consequences, and living with other adults who smoke tobacco. The wealth index, categorized into quintile rankings from one (lowest) to five (highest), was constructed using principal component analysis based on household assets such as electricity, cell telephone, car, and refrigerator^9,10^. Awareness of advertisements promoting tobacco products was defined as having seen or been exposed to advertisements promoting tobacco products, within the previous 30 days, in at least one location: stores selling cigarettes, television, radio, billboards, newspapers/magazines, the internet, public transportation, or on building walls. Severe health consequences included participants’ belief that smoking causes at least one of the following conditions: heart attack, cancer, stroke, or other serious illness. Living with other adults who smoke tobacco was defined based on the household questionnaire question: ‘Does this person currently smoke tobacco, including cigarettes, hand-rolled cigarettes, pipes, cigars and water pipes?’, which was asked for each adult living in the household. Year of the survey was included as a proxy for secular changes over time, including tobacco control measures and other environmental changes.

### Statistical analysis

Each round of GATS data was adjusted for non-response and weighted to represent the national adult population aged ≥15 years. Descriptive statistics, including point estimates and 95% confidence intervals (CI) for the unadjusted prevalence of current tobacco smoking and interest in quitting smoking were calculated, both overall and stratified by each characteristic of interest. Relative change, defined as percent change in the unadjusted prevalence between each pair of survey years was calculated [e.g. relative change between 2012 and 2016 = (GATS 2016 estimate – GATS 2012 estimate)/GATS 2012 estimate]. Chi-squared tests were used to assess significant differences (p<0.05) in the relative change in unadjusted prevalence between survey years.

Logistic regression analyses were conducted using pooled data of all three rounds of GATS to evaluate predictors of tobacco smoking and interest in quitting smoking. We estimated unadjusted (PR) and adjusted (APR) prevalence ratios and 95% confidence intervals for predictors of current tobacco smoking and interest in quitting smoking^11^. Adjusted models for each dependent variable included all *a priori* characteristics of interest.

Additionally, using multivariable logistic regression models that were stratified by each level of each characteristic of interest and adjusted for all other covariates, adjusted prevalence estimates and 95% confidence intervals were calculated; the marginal effects were calculated to represent the adjusted prevalence differences (APD) in the outcome measures between years. The unadjusted and adjusted PRs and APDs were considered as statistically significant if the two-side p value was <0.05.

All data preparation and analyses were conducted using SAS v9.4 and SAS-callable SUDAAN v11.0.1.

## Results

### Current tobacco use

Overall, the estimated prevalence and weighted number of adults who were classified as current tobacco smokers was 31.2% (95% CI: 30.0–32.6; n=16.0 million), 27.1% (95% CI: 25.8–28.3; n=14.8 million), and 31.6% (95% CI: 30.2–33.0; n=19.2 million) in 2008, 2012, and 2016, respectively (Table 1).

**Table 1:** Study population characteristics by survey year, prevalence of current tobacco smoking among adults aged ≥15 years, and the relative change in prevalence between survey years, GATS Turkey, 2008–2016

*Characteristics*	*Study population characteristics*			*Prevalence of tobacco smoking among adults*						*Relative change in prevalence of tobacco smoking^a^*		
	*2008*	*2012*	*2016*	*2008*		*2012*		*2016*		*2008–2012*	*2012–2016*	*2008–2016*
	*Weighted n (%)*	*Weighted n (%)*	*Weighted n (%)*	*%*	*95% CI*	*%*	*95% CI*	*%*	*95% CI*	*%*	*%*	*%*
**Overall**	51151089 (100)	54547734 (100)	60889089 (100)	31.2	30.0–32.6	27.1	25.8–28.3	31.6	30.2–33.0	-13.4*	16.7*	1.1
**Gender**												
Men	25095798 (49.1)	26861871 (49.2)	30328630 (49.8)	47.9	45.9–50.0	41.5	39.4–43.5	44.1	42.2–46.0	-13.5*	6.4	-8.0*
Women	26055290 (50.9)	27685862 (50.8)	30560459 (50.2)	15.2	14.0–16.5	13.1	12.0–14.3	19.2	17.5–21.0	-13.7*	46.4*	26.3*
**Age (years)**												
15–24	11523889 (22.5)	12205070 (22.4)	12989042 (21.3)	25.3	22.2–28.6	20.0	17.4–22.9	31.9	29.1–34.8	-20.8*	59.2*	26.1*
25–44	21843766 (42.7)	22902108 (42.0)	24685102 (40.5)	39.9	38.0–41.9	35.7	33.8–37.5	37.0	35.0–39.1	-10.7*	3.8	-7.3*
45–64	13097130 (25.6)	14283975 (26.2)	16563442 (27.2)	29.5	27.4–31.6	25.9	23.8–28.1	31.6	29.3–34.0	-12.1*	22.0*	7.3
≥65	4686303 (9.2)	5156580 (9.5)	6651503 (10.9)	10.3	8.5–12.4	8.8	7.2–10.7	10.9	8.8–13.5	-14.5	23.6	5.7
**Residence**												
Urban	35640186 (69.7)	39253547 (72.0)	56108840 (92.1)	33.0	31.4–34.7	29.0	27.4–30.7	32.1	30.6–33.6	-12.1*	10.6*	-2.7
Rural	15510902 (30.3)	15294186 (28.0)	4780248 (7.9)	27.2	25.3–29.1	22.0	20.4–23.8	25.5	22.3–29.1	-18.9*	16	-6
**Education level**												
No formal/less than primary	8719639 (17.1)	7484470 (13.7)	4173952 (6.9)	15.0	12.5–18.0	11.0	8.9–13.4	12.4	9.4–16.3	-27.0*	13.2	-17.3
Primary/less than secondary	24903381 (48.8)	24981205 (45.8)	23570315 (38.8)	31.4	29.5–33.2	26.5	24.7–28.3	27.3	25.5–29.1	-15.6*	3.0	-13.1*
Secondary or high school	12884850 (25.2)	15291240 (28.0)	24351818 (40.1)	42.0	39.4–44.6	36.1	33.7–38.6	37.3	35.2–39.4	-14.1*	3.3	-11.3*
College/university degree or higher	4564092 (8.9)	6787677 (12.4)	8663317 (14.3)	31.8	28.1–35.7	26.7	23.5–30.3	36.8	33.4–40.4	-15.8*	37.7*	15.9
**Wealth index**												
Quintile 1	7250570 (14.2)	7007069 (12.8)	3778797 (6.2)	28.0	25.1–31.0	21.2	18.3–24.3	27.2	22.9–32.1	-24.4*	28.8*	-2.7
Quintile 2	9388539 (18.4)	11094405 (20.3)	23902634 (39.3)	32.0	29.3–34.7	28.7	26.3–31.1	31.7	29.7–33.7	-10.3	10.5	-0.9
Quintile 3	11349646 (22.2)	14218395 (26.1)	10010179 (16.4)	32.5	29.7–35.4	28.3	26.1–30.5	33.5	30.6–36.4	-13.1*	18.5*	3.0
Quintile 4	13809332 (27.0)	14119564 (25.9)	15773723 (25.9)	32.2	30.1–34.4	27.2	25.0–29.5	32.1	29.7–34.7	-15.5*	18.0*	-0.2
Quintile 5	9353000 (18.3)	8108299 (14.9)	7423754 (12.2)	30.1	27.3–33.1	27.7	24.9–30.6	30.0	26.4–33.8	-8.1	8.3	-0.5
**Awareness of anti-tobacco warnings and advertisements on media^b^**												
Health warning labels on cigarette packages	41321765 (80.8)	46920235 (86.0)	46390020 (76.4)	36.5	35.1–38.0	29.7	28.3–31.0	34.5	32.8–36.1	-18.8*	16.1*	-5.7
Billboards	18392959 (36.0)	16313255 (29.9)	12915766 (21.3)	38.1	36.0–40.3	29.8	27.6–32.1	29.1	26.2–32.2	-21.8*	-2.3	-23.6*
Newspapers/magazines	23699834 (46.3)	22424225 (41.1)	16047183 (26.4)	36.1	34.2–38.1	29.5	27.7–31.4	31.9	29.1–34.9	-18.4*	8.3	-11.6*
Radio	11766346 (23.0)	13757629 (25.2)	12801505 (21.1)	33.8	31.0–36.9	30.5	28.1–33.0	28.8	25.9–32.0	-9.9	-5.5	-14.8*
Local television	43720452 (85.5)	49843373 (91.4)	44504458 (73.3)	32.1	30.7–33.5	27.1	25.9–28.5	32.0	30.4–33.7	-15.4*	18.0*	-0.2
**Awareness of any advertisements promoting tobacco products^c^**	3476260 (6.8)	5879116 (10.8)	7898853 (13.0)	34.4	29.7–39.3	27.5	23.9–31.4	35.2	31.2–39.3	-19.9*	27.8*	2.3
**Belief that smoking causes severe health consequences^d^**	49816619 (97.4)	54025753 (99.0)	60090701 (98.7)	31.3	30.0–32.6	27.0	25.8–28.3	31.5	30.1–32.9	-13.6*	16.6*	0.7
**Lives with other adults who smoke tobacco^e^**	9271531 (18.1)	23415665 (42.9)	22192587 (36.4)	45.0	41.4–48.8	32.9	30.9–35.0	39.1	36.7–41.5	-26.9*	18.7*	-13.2*

* Statistically significant (p<0.05 based on chi-squared test).

a Relative change calculated as the percent change in the unadjusted prevalence (e.g. relative change between 2012 and 2016 is equal to [GATS 2016 estimate - GATS 2012 estimate]/GATS 2012 estimate). The relative changes are calculated using un-rounded prevalence estimates and might be different if calculated using rounded prevalence estimates shown in this table.

b Categories are not mutually exclusive.

c Respondent reported awareness of advertisements promoting tobacco products within the previous 30 days in at least one of the following venues: stores selling cigarettes, television, radio, billboards, newspapers or magazines, the internet, public transportation, or building walls.

d Severe health consequences defined as heart attacks, cancer, stroke, or other serious illness.

e Tobacco products include cigarettes, hand-rolled cigarettes, pipes, cigars, and waterpipes. Includes daily, weekly, or monthly exposure to secondhand smoke in the home; adults who reported smoking in the home less frequently than monthly or smoking were not allowed in the home, were considered unexposed. Includes exposure within the previous 30 days; adults who did not use public transportation within the previous 30 days were considered unexposed.

In unadjusted analyses, a significant relative decrease in the prevalence of current tobacco smoking of >10% was observed for most demographic subgroups between 2008 and 2012, followed by varying significant increases in the prevalence between 2012 and 2016 (Table 1). Among men, the prevalence of current tobacco smoking significantly decreased by 13.5% between 2008 and 2012, from 47.9% (95% CI: 45.9–50.0) to 41.5% (95% CI: 39.4–43.5), with an overall relative decrease of 8% between 2008 and 2016 (44.1%, 95% CI: 42.2–46.0). Among women, a 13.7% decrease in the prevalence of current tobacco smoking between 2008 and 2012 was followed by a 46.4% relative increase between 2012 and 2016, resulting in an overall increase of approximately 26% between 2008 and 2016, from 15.2% (95% CI :14.0–16.5) to 19.2% (95% CI: 17.5–21.0). Statistically significant relative increases in the prevalence of current tobacco smoking were also noted among adults aged 15–24 years (26.1% increase) between 2008 and 2016. Significant relative decreases between 2008 and 2016 in the prevalence of current tobacco smoking were also noted among adults 25–44 years (7.3%), among those who completed primary or secondary/high school (13.1% and 11.3%, respectively), and among adults who were aware of anti-tobacco advertisements on billboards (23.6%), in newspapers or magazines (11.6%), or on the radio (14.8%), and among adults who lived with other adults who smoke tobacco (13.2%).

### Interest in quitting

The percentage and number of adults who currently smoke tobacco and were interested in quitting smoking decreased significantly from 53.0% (95% CI: 50.1–55.8; n=8.5 million) in 2008 to 32.8% (95% CI: 30.2–35.5; n=6.2 million) in 2016 (Table 2). Between 2008 and 2012, relative changes in the interest in quitting smoking varied by demographic subgroup, with significant decreases noted among those aged 15–24 years (14.4%), rural residents (2%), among those with college or higher levels of education (11.7%), those in the lowest and highest wealth quintiles (9.1% and 2.7%, respectively), and among those with awareness of anti-tobacco advertisements on billboards or in newspapers/magazines (2.7% and 1.1%, respectively). Between 2012 and 2016, and, overall, between 2008 and 2016, interest in quitting smoking among current smokers significantly declined by more than 20% in every demographic subgroup.

**Table 2:** Prevalence of any interest in quitting smoking among current tobacco smokers aged ≥15 years, and relative change in prevalence between survey years, GATS Turkey, 2008–2016

*Characteristics*	*Prevalence of any interest in quitting smoking among current tobacco smokers*						*Relative change in prevalence of any interest in quitting smoking^a^*		
	*2008*		*2012*		*2016*		*2008–2012*	*2012–2016*	*2008–2016*
	*%*	*95% CI*	*%*	*95% CI*	*%*	*95% CI*	*%*	*%*	*%*
**Overall**	53.0	50.1–55.8	55.2	52.3–58.0	32.8	30.2–35.5	4.2	-40.5*	-38.1*
**Gender**									
Men	53.6	50.6–56.5	53.8	50.6–57.0	33.9	31.2–36.7	0.5	-37.1*	-36.7*
Women	51.1	46.4–55.8	59.3	54.4–63.9	30.3	26.4–34.6	15.9*	-48.8*	-40.6*
**Age (years)**									
15–24	54.4	47.9–60.8	46.6	39.7–53.5	27.1	22.0–32.8	-14.4	-41.9*	-50.3*
25–44	52.4	48.8–56.0	57.6	54.0–61.2	32.5	29.5–35.6	10.0	-43.6*	-38.0*
45–64	54.0	49.7–58.3	55.1	50.9–59.2	37.8	33.4–42.3	2.0	-31.4*	-30.1*
≥65	45.4	35.2–56.1	57.6	46.8–67.7	33.1	22.6–45.7	26.7	-42.5*	-27.1*
**Residence**									
Urban	51.5	47.8–55.1	54.9	51.4–58.4	32.4	29.7–35.2	6.7	-41.0*	-37.1*
Rural	57.1	53.6–60.6	56.0	52.2–59.7	38.9	28.7–50.3	-2.0	-30.4*	-31.8*
**Education level**									
No formal/less than primary	41.4	32.7–50.6	45.5	35.6–55.8	22.5	13.1–35.9	10.0	-50.5*	-45.5*
Primary/less than secondary	51.8	48.2–55.4	54.4	50.2–58.5	33.3	29.7–37.1	5.0	-38.8*	-35.7*
Secondary or high school	54.6	50.3–58.8	57.2	53.1– 61.3	31.6	28.2– 35.2	4.9	-44.7*	-42.0*
College/university degree or higher	63.6	56.4–70.3	56.2	49.3– 62.8	36.8	30.5– 43.5	-11.7	-34.5*	-42.2*
**Wealth index**									
Quintile 1	52.5	46.6–58.3	47.7	41.1–54.3	34.2	25.8–43.8	-9.1	-28.2*	-34.7*
Quintile 2	47.3	41.6–53.1	54.4	49.1–59.6	30.2	26.8–33.9	15.0	-44.4*	-36.1*
Quintile 3	50.6	44.9–56.4	56.7	51.7–61.5	25.4	20.1–31.7	11.9	-55.1*	-49.8*
Quintile 4	55.4	50.8–59.9	56.0	50.9–61.1	43.3	38.6–48.1	1.2	-22.7*	-21.8*
Quintile 5	58.5	52.8–64.1	57.0	50.9–62.8	27.9	21.1–35.9	-2.7	-51.0*	-52.3*
**Awareness of anti-tobacco warnings and advertisements on media^b^**									
Health warning labels on cigarette packages	53.4	50.6–56.2	56.4	53.6–59.2	35.1	32.3–38.1	5.7	-37.8*	-34.2*
Billboards	56.6	52.5–60.6	55.1	50.3–59.7	35.2	30.1–40.6	-2.7*	-36.1*	-37.8*
Newspapers/magazines	55.7	52.0–59.4	55.1	50.9–59.2	34.6	30.1–39.4	-1.1*	-37.2*	-37.9*
Radio	56.6	51.5–61.7	58.1	53.1–63.1	34.0	28.9–39.5	2.7	-41.5*	-40.0*
Local television	54.2	51.3–57.2	56.6	53.7–59.4	34.7	31.8–37.7	4.4	-38.7*	-36.1*
**Awareness of any advertisements promoting tobacco products^c^**	58.4	49.2–67.1	60.2	50.7–69.0	31.3	25.6–37.7	3.0	-48.0*	-46.4*
**Belief that smoking causes severe health consequences^d^**	53.4	50.6–56.2	55.6	52.7–58.4	33.1	30.5–35.8	4.0	-40.4*	-38.0*
**Lives with other adults who smoke tobacco^e^**	51.6	45.9–57.3	53.4	49.7–57.2	32.1	28.5–36.1	3.5	-39.9*	-37.7*

* Statistically significant (p<0.05 based on chi-squared test).

a Relative change calculated as the percent change in the unadjusted prevalence (e.g. relative change between 2012 and 2016 is equal to [GATS 2016 estimate - GATS 2012 estimate]/GATS 2012 estimate). The relative changes are calculated using un-rounded prevalence estimates and might be different if calculated using rounded prevalence estimates shown in this table.

b Categories are not mutually exclusive.

c Respondent reported awareness of advertisements promoting tobacco products within the previous 30 days in at least one of the following venues: stores selling cigarettes, television, radio, billboards, newspapers or magazines, the internet, public transportation, or building walls.

d Severe health consequences defined as heart attacks, cancer, stroke, or other serious illness.

e Tobacco products include cigarettes, hand-rolled cigarettes, pipes, cigars, and waterpipes.

### Multivariate analyses

Adjusted for all other variables, the prevalence of tobacco smoking was significantly lower in 2012 (APR=0.77; 95% CI: 0.72–0.81) and in 2016 (APR=0.90; 95% CI: 0.85–0.96) than in 2008, among women compared to men (APR=0.38; 95% CI: 0.36–0.40), among residents of rural areas compared to their urban counterparts (APR=0.86; 95% CI: 0.81–0.91), among those in the highest two wealth quintiles compared to those in the lowest wealth quintile (Quintile 4: APR=0.90; 95% CI: 0.83–0.97; Quintile 5: APR=0.88; 95% CI: 0.80–0.96), among those that were aware of anti-tobacco advertisements on local television (APR=0.93; 95% CI 0.87–1.00), and among those that believed smoking causes severe health consequences compared to those who did not (APR=0.69; 95% CI: 0.60–0.79) (Table 3). The adjusted prevalence of tobacco smoking was higher among survey respondents with higher levels of education than no formal schooling/less than primary (primary/less than secondary: APR=1.15; 95% CI: 1.04–1.28; secondary or high school: APR=1.36; 95% CI: 1.22–1.52; college/university: APR=1.16; 95% CI: 1.02–1.31), among those who were aware of health warning labels on cigarette packages compared to those who did not (APR=1.58; 95% CI: 1.45–1.73), and among those that lived with other adults who smoke tobacco (APR=1.55; 95% CI: 1.49–1.62). Compared to young adults aged 15–24 years, adults aged 25–44 years (APR=1.63; 95% CI: 1.51–1.74) or 45–64 years (APR=1.36; 95% CI: 1.25–1.47) were more likely to smoke tobacco, while those ≥65 years were less likely to smoke tobacco (APR=0.63; 95% CI: 0.55–0.72).

**Table 3:** Unadjusted and adjusted prevalence ratios for predictors of current tobacco smoking and any interest in quitting tobacco smoking, GATS Turkey, 2008–2016

*Characteristics*	*Current tobacco smoking*				*Any interest in quitting tobacco smoking^a^*			
	*PR^b^*	*95% CI*	*APR^c^*	*95% CI*	*PR^b^*	*95% CI*	*APR^c^*	*95% CI*
**Year of survey**								
2008 (Ref.)	1		1		1		1	
2012	0.87	0.81–0.92*	0.77	0.72–0.81*	1.04	0.97–1.12	1.04	0.96–1.12
2016	1.01	0.95–1.07	0.90	0.85–0.96*	0.62	0.56– 0.68*	0.66	0.60–0.73*
**Gender**								
Men (Ref.)	1		1		1		1	
Women	0.36	0.34–0.38*	0.38	0.36–0.40*	0.95	0.90–1.01	1.01	0.95–1.07
**Age (years)**								
15–24 (Ref.)	1		1		1		1	
25–44	1.45	1.35–1.56*	1.63	1.51–1.74*	1.16	1.05–1.28*	1.08	0.99–1.19
45–64	1.13	1.04–1.22*	1.36	1.25–1.47*	1.18	1.06–1.30*	1.14	1.03–1.25*
≥65	0.39	0.34–0.45*	0.63	0.55–0.72*	1.07	0.90–1.28	1.13	0.96–1.32
**Residence**								
Urban (Ref.)	1		1		1		1	
Rural	0.79	0.74–0.83*	0.86	0.81–0.91*	1.23	1.15–1.31*	1.13	1.05–1.22*
**Education level**								
No formal/less than primary (Ref.)	1		1		1		1	
Primary/less than secondary	2.18	1.92–2.47*	1.15	1.04–1.28*	1.20	1.02–1.41*	1.21	1.02–1.43*
Secondary or high school	2.93	2.57–3.33*	1.36	1.22–1.52*	1.15	0.98–1.35	1.25	1.05–1.48*
College/university degree or higher	2.48	2.16–2.85*	1.16	1.02–1.31*	1.24	1.03–1.48*	1.36	1.13–1.63*
**Wealth index**								
Quintile 1 (Ref.)	1		1		1		1	
Quintile 2	1.23	1.13–1.34*	0.99	0.92–1.07	0.84	0.76–0.94*	0.96	0.86–1.07
Quintile 3	1.23	1.13–1.35*	1.00	0.92–1.08	0.97	0.87–1.08	0.98	0.88–1.10
Quintile 4	1.21	1.11–1.33*	0.90	0.83–0.97*	1.09	0.99–1.20	1.10	0.99–1.23
Quintile 5	1.16	1.06–1.28*	0.88	0.80–0.96*	1.04	0.92–1.17	1.03	0.91–1.17
**Awareness of anti-tobacco warnings and advertisements on media^d^**								
Health warning labels on cigarette packages	2.16	1.95–2.39*	1.58	1.45–1.73*	1.71	1.44–2.03*	1.30	1.12–1.51*
Billboards	1.14	1.08–1.20*	1.00	0.95–1.06	1.17	1.09–1.25*	1.02	0.95–1.09
Newspapers/magazines	1.15	1.09–1.21*	0.99	0.94–1.05	1.17	1.10–1.24*	0.99	0.92–1.06
Radio	1.04	0.98–1.10	0.97	0.91–1.03	1.12	1.05–1.21*	1.02	0.94–1.09
Local television	1.05	0.98–1.13	0.93	0.87–1.00*	1.46	1.29–1.65*	1.16	1.04–1.30*
**Awareness of any advertisements promoting tobacco products^e^**	1.09	1.00–1.18*	1.01	0.94–1.10	0.99	0.88–1.12	1.06	0.96–1.17
**Belief that smoking causes severe health consequences^f^**	0.87	0.74–1.02	0.70	0.61–0.80*	1.80	1.30–2.48*	1.57	1.17–2.11*
**Lives with other adults who smoke tobacco^g^**	1.42	1.36–1.49*	1.55	1.49–1.62*	0.93	0.87–1.00*	0.97	0.91–1.03

PR: prevalence ratio. APR: adjusted prevalence ratio.

* Statistically significant (p<0.05).

a Among current tobacco smokers.

b Calculated as the ratio of average marginal predictions in logistic regression models that accounted for the complex survey design but were unadjusted for model covariates.

c Calculated as the ratio of average marginal predictions in multivariate logistic regression models that accounted for the complex survey design and adjusted for all model covariates.

d Variables are not mutually exclusive. Reference value is no report of awareness for the specified type of anti-tobacco warning or advertisement.

e Respondent reported awareness of advertisements promoting tobacco products within the previous 30 days in at least one of the following venues: stores selling cigarettes, television, radio, billboards, newspapers or magazines, the internet, public transportation, or building walls.

f Severe health consequences defined as heart attacks, cancer, stroke, or other serious illness.

g Tobacco products include cigarettes, hand-rolled cigarettes, pipes, cigars, and waterpipes.

In models assessing predictors of interest in quitting smoking among adults who currently smoke tobacco, there was no significant difference between 2008 and 2012, but the prevalence of interest in quitting smoking was significantly lower in 2016 than in 2008 (APR=0.66; 95% CI: 0.60–0.73) (Table 3). Other significant predictors of any interest in quitting smoking included age, with an increase in interest noted among persons 45–64 years compared to persons aged 15–24 years (APR=1.14; 95% CI: 1.03–1.25), living in a rural area (APR=1.13; 95% CI: 1.05– 1.22), completion of primary school or higher levels of education (primary: APR=1.21; 95% CI: 1.02–1.43; secondary: APR=1.25 (95% CI: 1.05–1.48; college or higher: APR=1.36; 95% CI: 1.13–1.63), awareness of anti-tobacco warnings on cigarette packages (APR=1.30; 95% CI: 1.12–1.51) and on television (APR=1.16; 95% CI: 1.04–1.30), and a belief that smoking causes severe health consequences (APR=1.57; 95% CI: 1.17–2.11).

Model-adjusted prevalence estimates and differences in the model-adjusted prevalence of current tobacco smoking between survey years are presented in Table 4. Overall, the model-prevalence of tobacco smoking was 33.9% (95% CI: 32.6–35.4) in 2008, 26.1% (95% CI: 24.9–27.3) in 2012, and 30.6% (95% CI: 29.2–30.2) in 2016. We observed a significant 7.9% (95% CI: -9.6 – -6.1) decrease in the prevalence of current tobacco smoking between 2008 and 2012 and a 4.5% (95% CI: 2.8–6.3) significant increase between 2012 and 2016, with a significant decrease between 2008 and 2016 (adjusted prevalence difference, APD= -3.3%; 95% CI: -5.3 – -1.4). A similar pattern of change was observed among most subgroups in the analysis.

**Table 4:** Model-adjusted prevalence of current tobacco smoking and pairwise comparisons of the difference in the stratified, model-adjusted prevalence between survey years, GATS Turkey, 2008–2016

*Characteristics*	*Model-adjusted prevalence of current tobacco smoking^a^*						*Difference in model-adjusted prevalence of current tobacco smoking^a^*					
	*2008*		*2012*		*2016*		*2008–2012*		*2012–2016*		*2008–2016*	
	*%*	*95% CI*	*%*	*95% CI*	*%*	*95% CI*	*%*	*95% CI*	*%*	*95% CI*	*%*	*95% CI*
**Overall**	33.9	32.6–35.4	26.1	24.9–27.3	30.6	29.2–32.0	-7.9	-9.6 – -6.1*	4.5	2.8–6.3*	-3.3	-5.3 – -1.4*
**Gender**												
Men	47.9	45.8–50	40.3	38.3–42.3	45.3	43.3–47.4	-7.6	-10.4 – -4.8*	5.1	2.2–7.9*	-2.5	-5.6 – 0.5*
Women	21.1	19.4–22.8	12.1	11.1–13.2	16.2	14.7–17.8	-8.9	-10.8 – -7.1*	4.1	2.3–5.9*	-4.9	-7.1 – -2.6*
**Age (years)**												
15–24	27.6	24.4–31	20.7	18.0–23.7	29.1	26.3–32.1	-6.9	-10.9 – -2.8*	8.4	4.3–12.5*	1.5	-3.0 – 6.1
25–44	44.2	42.0–46.4	34.1	32.4–35.8	35.4	33.4–37.4	-10.1	-12.8 – -7.4*	1.3	-1.3 – 3.9	-8.8	-11.8 – -5.8*
45–64	32.5	30.3–34.8	24.7	22.8–26.7	30.5	28.3–32.8	-7.8	-10.6 – -4.9*	5.8	2.8–8.9*	-2.0	-5.2 – 1.3
≥65	10.7	8.7–13.1	8.1	6.5–9.9	11.7	9.4–14.5	-2.6	-5.3 – 0.0*	3.6	0.5–6.8	1.0	-2.5 – 4.6
**Residence**												
Urban	35.7	34.0–37.5	27.4	26.0–28.9	31.9	30.4–33.4	-8.3	-10.5 – -6.0*	4.4	2.4–6.4*	-3.8	-6.1 – -1.5*
Rural	27.7	25.9–29.6	21.7	20.2–23.3	25.9	22.5–29.7	-6.0	-8.3 – -3.7*	4.2	0.2–8.3*	-1.8	-5.8 – 2.3
**Education level**												
No formal/less than primary	16.5	13.9– 19.5	9.7	8.1–11.7	13.5	10.8–16.7	-6.8	-9.8 – -3.8*	3.8	0.5–7.0*	-3.0	-7.0 – 1.0
Primary/less than secondary	31.6	29.8–33.5	25.7	24.1–27.4	27.9	26.0–29.9	-5.9	-8.3 – -3.5*	2.2	-0.3 – 4.7	-3.7	-6.4 – -1.0*
Secondary or high school	43.6	40.9–46.3	34.0	31.8–36.3	37.9	35.8–40.0	-9.6	-13 – -6.2*	3.8	0.8–6.8*	-5.7	-9.2 – -2.2*
College/university degree or higher	34.8	30.9–39	25.6	22.5–28.9	36.4	33.1–39.8	-9.2	-14.1 – -4.4*	10.8	6.1–15.4*	1.5	-3.8 – 6.8
**Wealth index**												
Quintile 1	30.0	27.0–33.1	21.1	18.3–24.2	24.6	20.7–29.0	-8.9	-12.6 – -5.1*	3.5	-1.8 – 8.8	-5.4	-10.5 – -0.2*
Quintile 2	35.5	32.5–38.7	26.7	24.6–29.0	31.6	29.7–33.5	-8.8	-12.4 – -5.1*	4.8	1.9–7.8*	-3.9	-7.6 – -0.3*
Quintile 3	34.2	31.3–37.2	26.7	24.8–28.8	34.2	31.3–37.3	-7.5	-11.0 – -4.0*	7.5	3.9–11.1*	0.0	-4.2 – 4.3
Quintile 4	35.9	33.6–38.2	26.9	24.7–29.3	29.4	27.2–31.8	-8.9	-12.1 – -5.8*	2.5	-0.7 – 5.7	-6.5	-9.6 – -3.3*
Quintile 5	31.6	28.7–34.6	26.6	24.1–29.4	29.5	26.1–33.2	-4.9	-8.8 – -1.0*	2.9	-1.5 – 7.3	-2.0	-6.6 – 2.5
**Awareness of anti-tobacco warnings and advertisements on media^b^**												
Health warning labels on cigarette packages	38.7	37.2–40.4	29.3	28.1–30.6	33.1	31.5–34.7	-9.4	-11.4 – -7.4*	3.8	1.8–5.8*	-5.7	-7.9 – -3.4*
Billboards	40.5	38.2–42.7	28.8	26.7–30.9	27.6	24.8–30.6	-11.7	-14.7 – -8.7*	-1.2	-4.8 – 2.5	-12.9	-16.6 – -9.2*
Newspapers/magazines	38.1	36.1–40.1	28.8	27.0–30.5	30.6	27.8–33.4	-9.3	-11.9 – -6.7*	1.8	-1.6 – 5.2	-7.5	-10.9 – -4.1*
Radio	38.6	35.7–41.7	29.6	27.3–32.1	26.2	23.5–29.1	-9.0	-12.7 – -5.3*	-3.4	-7.2 – 0.3*	-12.4	-16.4 – -8.4*
Local television	35.2	33.7–36.8	26.5	25.3–27.7	30.2	28.6–31.8	-8.7	-10.6 – -6.8*	3.7	1.7–5.7*	-5.0	-7.2 – -2.8*
**Awareness of any advertisements promoting tobacco product^c^**	35.5	30.8–40.5	26.1	22.6–30.0	35.9	32.1–39.9	-9.4	-15.3 – -3.5*	9.8	4.5–15.1*	0.4	-5.9 – 6.6
**Belief that smoking causes severe health consequences^d^**	34.0	32.6–35.4	26.0	24.8–27.2	30.5	29.1–31.9	-8.0	-9.7 – -6.2*	4.5	2.7–6.3*	-3.5	-5.4 – -1.5*
**Lives with other adults who smoke tobacco^e^**	39.9	36.3–43.5	34.9	32.8–37.1	39.1	36.7–41.5	-5.0	-9.1 – -0.8*	4.2	1.0–7.3*	-0.8	-5.2 – 3.7

* Statistically significant (p<0.05).

a Calculated as the marginal effect in multivariate logistic regression models that were stratified by each level of each characteristic and adjusted for all other covariates in the table.

b Variables are not mutually exclusive. Reference value is no report of awareness for the specified type of anti-tobacco warning or advertisement.

c Respondent reported awareness of advertisements promoting tobacco products within the previous 30 days in at least one of the following venues: stores selling cigarettes, television, radio, billboards, newspapers or magazines, the internet, public transportation, or on building walls.

d Severe health consequences defined as heart attacks, cancer, stroke, or other serious illness.

e Tobacco products include cigarettes, hand-rolled cigarettes, pipes, cigars, and waterpipes.

Among adults who currently smoke tobacco, there was little change in interest in quitting smoking between 2008 and 2012, but a significant decline in interest between 2012 and 2016 in all subgroups (Table 5). Overall, the model-adjusted prevalence of interest in quitting smoking was 52.0% (95% CI: 49.0–55.0) in 2008, 53.9% (95% CI: 51.0–56.7) in 2012, and 34.5% (95% CI: 31.7–37.3) in 2016. We observed significantly less overall interest in quitting smoking in 2016 than in 2008 (APD= -17.5%; 95% CI: -21.8 – -13.4).

**Table 5:** Model-adjusted prevalence of any interest in quitting tobacco smoking among current tobacco smokers and pairwise comparisons of the difference in the stratified, model-adjusted prevalence between survey years, GATS Turkey, 2008–2016

*Characteristics*	*Model-adjusted prevalence of any interest in quitting tobacco smoking*						*Difference in model-adjusted prevalence of any interest in quitting tobacco smoking^a,b^*					
	*2008*		*2012*		*2016*		*2008–2012*		*2012–2016*		*2008–2016*	
	*%*	*95% CI*	*%*	*95% CI*	*%*	*95% CI*	*%*	*95% CI*	*%*	*95% CI*	*%*	*95% CI*
**Overall**	52.0	49.0–55.0	53.9	51.0–56.7	34.5	31.7–37.3	1.9	-2.2–6.0	-19.4	-23.5 – -15.3*	-17.5	-21.8 – -13.3*
**Gender**												
Men	52.5	49.4–55.6	53.1	49.8–56.3	35.4	32.5–38.5	0.6	-3.8 – 5.0	-17.7	-22.3 – -13.1*	-17.1	-21.6 – -12.5*
Women	52.2	46.5–57.8	56.2	51.1–61.0	31.3	27.2–35.8	4.0	-3.5 – 11.4	-24.8	-31.8 – -17.9*	-20.9	-28.4 – -13.4*
**Age (years)**												
15–24	53.8	46.9–60.6	46.5	39.5–53.7	27.2	21.8–33.4	-7.3	-16.9 – 2.3	-19.3	-29.0 – -9.6*	-26.6	-36.1– -17.1*
25–44	52.0	48.1–55.9	56.9	53.2–60.5	33.5	30.3–36.8	4.9	-0.4 – 10.2	-23.4	-28.4 – -18.4*	-18.6	-24.0 – -13.2*
45–64	53.3	48.6–57.9	53.1	48.7–57.5	39.9	35.2–44.7	-0.1	-6.5 – 6.2	-13.2	-19.9 – -6.6*	-13.4	-20.4 – -6.4*
≥65	45.3	34.4–56.5	52.3	40.8–63.6	36.0	25.3–48.3	7.1	-6.5 – 20.7	-16.3	-33.5 – 0.9	-9.2	-26.1 – 7.6
**Residence**												
Urban	50.6	46.8–54.4	53.7	50.3–57.2	33.6	30.8–36.4	3.1	-2.0 – 8.2	-20.2	-24.7 – -15.6*	-17.0	-21.8 – -12.3*
Rural	57.5	53.9–61.1	55.7	51.8–59.4	38.9	29.9–48.8	-1.8	-7.1 – 3.5	-16.7	-26.9 – -6.6*	-18.6	-28.6 – -8.6*
**Education level**												
No formal/less than primary	41.8	32.3–51.9	44.2	35.3–53.6	23.3	13.4–37.5	2.5	-10.4 – 15.3	-20.9	-36.0 – -5.8*	-18.4	-35.6 – -1.3*
Primary/less than secondary	51.2	47.4–55.0	53.6	49.5–57.6	34.8	30.8–39.0	2.4	-3.1 – 7.8	-18.8	-24.7 – -12.9*	-16.4	-22.2 – -10.6*
Secondary or high school	52.2	47.6–56.7	55.0	50.7–59.2	34.1	30.5–37.9	2.8	-3.3 – 9.0	-20.9	-26.8 – -14.9*	-18.0	-24.0 – -12.1*
College/university degree or higher	59.8	51.5–67.7	54.2	47.2–61.0	39.4	33.1–46.1	-5.6	-16 – 4.8	-14.8	-24.3 – -5.3*	-20.4	-31.1 – -9.8*
**Wealth index**												
Quintile 1	52.2	45.8–58.4	46.2	39.5–53.0	37.3	27.8–47.8	-6.0	-14.6 – 2.7	-9.0	-22.1 – 4.2	-14.9	-27.8 – -2.0*
Quintile 2	44.6	38.7–50.6	52.8	47.5–58.0	31.8	28.3–35.5	8.2	0.7 – 15.7*	-21.0	-27.5 – -14.4*	-12.8	-19.9 – -5.7*
Quintile 3	50.9	44.8–57.0	54.6	49.5–59.6	27.1	21.4–33.7	3.7	-4.2 – 11.6	-27.5	-35.4 – -19.6*	-23.7	-32.6 – -14.8*
Quintile 4	55.9	51.0–60.7	55.0	49.8–60.2	43.7	38.8–48.8	-0.9	-7.9 – 6.2	-11.3	-18.7 – -4*	-12.2	-19.4 – -5.0*
Quintile 5	57.8	51.5–63.9	56.0	49.7–62.1	29.2	22.7–36.7	-1.8	-10.7 – 7.0	-26.8	-36.0 – -17.6*	-28.7	-38.1 – -19.2*
**Awareness of anti-tobacco warnings and advertisements on media^c^**												
Health warning labels on												
cigarette packages	53.3	50.3–56.3	56.0	53.1–58.8	35.6	32.7–38.7	2.7	-1.5 – 6.8	-20.3	-24.6 – -16.1*	-17.7	-22.0 – -13.3*
Billboards	57.8	53.5–62.0	54.9	50.0–59.6	33.3	28.5–38.5	-3.0	-9.4 – 3.5	-21.6	-28.6 – -14.5*	-24.5	-31.3 – -17.7*
Newspapers/magazines	55.5	51.5–59.4	54.7	50.4–58.9	35.6	31.0–40.5	-0.8	-6.6 – 5.0	-19.1	-25.6 – -12.5*	-19.9	-26.3 – -13.5*
Radio	57.7	52.2–63.0	57.5	52.2–62.6	33.6	28.7–39.0	-0.2	-7.8 – 7.4	-23.9	-31.7 – -16.1*	-24.1	-31.8 – -16.3*
Local television	54.3	51.1–57.4	56.6	53.7–59.5	34.7	31.7–37.8	2.4	-1.9 – 6.6	-21.9	-26.2 – -17.7*	-19.6	-24.1 – -15.0*
**Awareness of any advertisements promoting tobacco products^d^**	58.4	49.0–67.2	59.4	50.2–68.0	32.0	26.3–38.2	1.0	-11.0 – 13.0	-27.4	-38.0 – -16.9*	-26.4	-37.4 – -15.4*
**Belief that smoking causes severe health consequences^e^**	52.6	49.5–55.6	54.7	51.8–57.6	34.3	31.6–37.2	2.2	-2.0 – 6.3	-20.4	-24.5 – -16.2*	-18.2	-22.5 – -14.0*
**Lives with other adults who smoke tobacco^f^**	53.6	47.6–59.6	52.2	48.3–56.0	32.3	28.5–36.3	-1.5	-8.5 – 5.6	-19.9	-25.5 – -14.3*	-21.4	-28.8 – -14.0*

a Calculated as the marginal effect in multivariate logistic regression models that were stratified by each level of each characteristic and adjusted for all other covariates in the table.

b Among current smokers.

c Variables are not mutually exclusive. Reference value is no report of awareness for the specified type of anti-tobacco warning or advertisement.

d Respondent reported awareness of advertisements promoting tobacco products within the previous 30 days in at least one of the following venues: stores selling cigarettes, television, radio, billboards, newspapers or magazines, the internet, public transportation, or on building walls.

e Severe health consequences defined as heart attacks, cancer, stroke, or other serious illness.

f Tobacco products include cigarettes, hand-rolled cigarettes, pipes, cigars, and waterpipes.

## DISCUSSION

Although the prevalence of current tobacco smoking in Turkey decreased significantly in nearly every demographic subgroup between 2008 and 2012, data from GATS indicate that the prevalence of current tobacco smoking had largely returned to 2008 levels by 2016. Moreover, a significant decline in interest in quitting smoking among current tobacco smokers was noted among men and women and adults aged <65 years between 2008 and 2016, particularly between 2012 and 2016.

The decrease in current tobacco smoking prevalence in Turkey between 2008 and 2012 was largely attributed to the implementation of tobacco control policies and their national action plan^4^. In 2008, legislation was implemented that prohibited almost all types of tobacco advertising in Turkey and fines were introduced for individuals who smoke in areas where smoking is prohibited^12^. Law Number 4207 was revised in 2009 to expand the prohibition of tobacco smoking to include most indoor public places that were not already prohibited in 1997 (e.g. restaurants and bars)^12^. Tobacco excise taxes also increased during this period, to the current rate of approximately 66% of the retail price of cigarettes, which effectively more than doubled the price of manufactured cigarettes between 2002 and 2011^1,12^. In 2013, WHO recognized Turkey as the first country with all MPOWER measures at the highest level^13^.

However, despite nearly doubling the mean cost of a pack of manufactured cigarettes by 2012 and adopting comprehensive tobacco control policies^2^, the prevalence of tobacco smoking increased between 2012 and 2016. This might be attributed, in part, to the income gains during this period, as indicated by increases in Turkey’s per capita Gross Domestic Product^14^; tobacco excise taxes were also lower than the target of 70% of retail prices recommended by WHO^12^. In addition, mid-priced tobacco product availability and marketing of these products increased options as well as popularity, as some consumers may have shifted from premium brands to more economical brands of manufactured cigarettes^15^. Other factors that have been noted as barriers to decreasing tobacco use are lack of enforcement of legislation banning smoking in most public spaces and lack of legislation banning retail product displays^12,15^. Although advertisements promoting tobacco products have been prohibited in most venues in Turkey for several years, such exposure was not uncommon among GATS respondents, with approximately 13% of respondents in the 2016 survey reporting awareness of advertisements in the previous 30 days. Lastly, the tobacco landscape continues to change with availability of new products presenting ongoing challenges for Turkey, including enforcement of existing laws^16^.

This study found that interest in quitting smoking decreased among most demographic subgroups between 2008 and 2016, which may be due, in part, to lower awareness of anti-tobacco warnings and advertisements and health outcomes and may also reflect the high level of social acceptance of tobacco smoking in Turkey, where nearly half of adult men and one in five women reported currently smoking tobacco in 2016. However, this study also identified potential opportunities for interventions, including continued implementation of a comprehensive approach using MPOWER strategies to reduce smoking and increase interest in quitting smoking. For example, the belief that tobacco smoking causes severe health consequences was associated with a significantly lower prevalence of current tobacco smoking and was also associated with an increased interest in quitting smoking. This finding suggests that messages about serious adverse health outcomes related to tobacco smoking in specific types of channels may be an important element to highlight in anti-tobacco advertisements aimed at preventing initiation of tobacco smoking or increasing interest in smoking cessation.

Additionally, in adjusted analyses, residents of urban areas were significantly more likely to smoke tobacco and significantly less likely to have interest in quitting smoking. There was a lower prevalence of current tobacco smoking among respondents with awareness of anti-tobacco warnings and advertisements on local television compared to those who were not, and awareness of televised anti-tobacco advertisements was significantly associated with interest in quitting. Lastly, although the prevalence of current tobacco smoking in Turkey was lower among young adults aged 15–24 years than among adults aged 25–64 years, we observed the largest increase in prevalence from 2012 to 2016 in those aged 15–24 years. Earlier initiation of smoking has been associated with longer duration of smoking and increased likelihood of nicotine dependence^17^.

Turkey has demonstrated their commitment to monitoring and surveillance of tobacco use in several ways. First, they conducted multiple rounds of GATS, the Global Youth Tobacco Survey, and other national surveys^3,13,18^ to continue to monitor tobacco prevalence and other important tobacco use and behavior metrics. In 2015, Turkey published their National Tobacco Control Program Plan of Action (2015–2018), which outlined a number of strategies and activities to meet several tobacco control and cessation goals, such as increasing public awareness of the harmful effects of tobacco use, increase the rate of smoking cessation to >50%, and to completely eliminate all forms of tobacco advertising^12^. Additional ongoing or planned tobacco control measures in Turkey include government support of the ALO 171 Quitline, including revisions to scripts targeted to callers aged <18 years, support for smoking cessation clinics and providing cessation medications free of charge, increased enforcement and monitoring of smoke-free policies, anti-tobacco television ads, prevention of the import and marketing of electronic cigarettes and heated tobacco products, and implementation of plain cigarette packaging^12,15^.

### Strengths and limitations

The analysis was strengthened by use of GATS data, which are nationally representative and utilize standardized protocols and procedures. Using standard protocols and procedures increases the reliability of comparing estimates over time and with other countries. However, despite these strengths, the study is still subject to at least four limitations. First, this study did not assess the number and distribution of television channels with anti-tobacco media and/or content of the anti-tobacco media, which may vary depending on geographical area or among channels preferred by different population subgroups. Second, GATS did not collect information on internet or social media usage throughout the study period, so we could not assess the impact of these sources of tobacco promotion or anti-tobacco advertising on the prevalence of current tobacco smoking or interest in quitting smoking or respondent ability to purchase products online such as heated tobacco products and electronic cigarettes. The third limitation of this study was the use of self-reported survey data, which may have introduced social desirability reporting bias resulting in artificially lower prevalence of tobacco smoking or higher interest in quitting smoking. However, as tobacco smoking is relatively common in Turkey, social acceptance of smoking is also relatively high, and therefore unlikely to have substantially impacted the findings. Lastly, the data for this study are from repeated cross-sectional surveys; while these data can be used to analyze population-level changes over time, they cannot be used to assess individual-level changes.

## Conclusions

While Turkey demonstrated progress in decreasing current tobacco smoking between 2008 and 2012, results from GATS 2016 indicate that this progress was not sustained, and current tobacco smoking returned to 2008 levels. In addition, between 2008 and 2016, a significant decline in interest in quitting smoking among current tobacco smokers was observed. However, this analysis also highlighted opportunities for targeted interventions aimed at reducing tobacco smoking and increasing interest in quitting smoking as well as Turkey’s ongoing commitment to tobacco control and prevention. These opportunities can be further strengthened by implementation and enforcement of the evidence-based measures in Turkey’s National Action Plans.

## Data Availability

The data supporting this research are available from the GTSSData at https://nccd.cdc.gov/GTSSDataSurveyResources/Ancillary/DataReports.aspx?CAID=2
